# Role of Hfq in Genome Evolution: Instability of G-Quadruplex Sequences in *E. coli*

**DOI:** 10.3390/microorganisms8010028

**Published:** 2019-12-22

**Authors:** Virali J. Parekh, Brittany A. Niccum, Rachna Shah, Marisa A. Rivera, Mark J. Novak, Frederic Geinguenaud, Frank Wien, Véronique Arluison, Richard R. Sinden

**Affiliations:** 1 Laboratory of DNA Structure and Mutagenesis, Department of Chemistry and Applied Biological Sciences, South Dakota School of Mines and Technology, Rapid City, SD 57701, USA; virali.parekh@mines.sdsmt.edu; 2 Department of Mathematics, Florida Institute of Technology, Melbourne, FL 32901, USA; brittanyniccum@gmail.com; 3 Department of Biological Sciences, Florida Institute of Technology, Melbourne, FL 32901, USA; rachnashah84@gmail.com (R.S.); riveram818@gmail.com (M.A.R.); 4 Department of Chemistry and Applied Biological Sciences, South Dakota School of Mines and Technology; Rapid City, SD 57701, USA; mark.novak1990@gmail.com; 5 Plateforme CNanoMat & Inserm U1148, Laboratory for Vascular Translational Science, UFR SMBH, Université Paris 13, Sorbonne Paris Cité, F-93017 Bobigny, France; frederic.geinguenaud@univ-paris13.fr; 6 Synchrotron SOLEIL, 91192 Gif-sur-Yvette, France; frank.wien@synchrotron-soleil.fr; 7 Laboratoire Léon Brillouin LLB, CEA, CNRS UMR12, Université Paris Saclay, CEA Saclay, 91191 Gif-sur-Yvette, France; 8 Université de Paris, UFR Sciences du vivant, 35 rue Hélène Brion, 75205 Paris cedex, France

**Keywords:** genomic instability, quadruplex, DNA, mutagenesis, nucleoid, bacterial chromatin

## Abstract

Certain G-rich DNA repeats can form quadruplex in bacterial chromatin that can present blocks to DNA replication and, if not properly resolved, may lead to mutations. To understand the participation of quadruplex DNA in genomic instability in *Escherichia coli* (*E. coli*), mutation rates were measured for quadruplex-forming DNA repeats, including (G_3_T)_4_, (G_3_T)_8_, and a RET oncogene sequence, cloned as the template or nontemplate strand. We evidence that these alternative structures strongly influence mutagenesis rates. Precisely, our results suggest that G-quadruplexes form in *E. coli* cells, especially during transcription when the G-rich strand can be displaced by R-loop formation. Structure formation may then facilitate replication misalignment, presumably associated with replication fork blockage, promoting genomic instability. Furthermore, our results also evidence that the nucleoid-associated protein Hfq is involved in the genetic instability associated with these sequences. Hfq binds and stabilizes G-quadruplex structure in vitro and likely in cells. Collectively, our results thus implicate quadruplexes structures and Hfq nucleoid protein in the potential for genetic change that may drive evolution or alterations of bacterial gene expression.

## 1. Introduction

**Figure 1:**
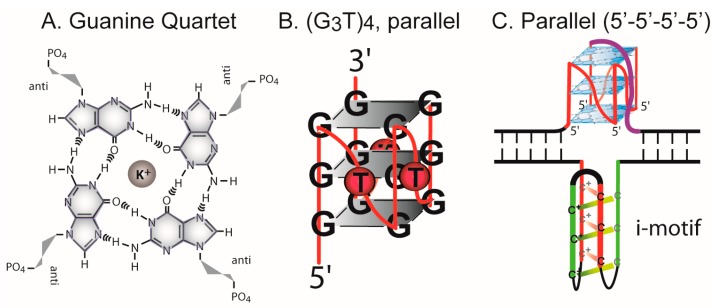
G-quadruplex and i-motif structures. (**A**) G_4_ guanine quartet with sugars in the anti configuration and a stabilizing K^+^ ion. (**B**) (G_3_T)_4_ parallel quadruplex. (**C**) A parallel G-quadruplex in duplex DNA opposite an i-motif.

Many DNA repeat motifs, common in the human genome, can adopt noncanonical DNA conformations, including cruciforms, left-handed Z-DNA, intramolecular triplex DNA, and slipped strand structures [1]. A guanine quadruplex quartet containing Hoogsteen hydrogen bonds (Figure 1A) [2], is a basic component of a myriad of remarkably stable four-stranded structures formed from tracts of three or more guanines separated by one or more intervening bases (Figure 1B). Stacked quartets are stabilized by monovalent cations. The topology of quadruplex structures is highly variable with strands arranged in a parallel, antiparallel, or mixed orientations associated with various glycosidic configurations of guanines [1,3,4,5]. A single repeat motif can often form multiple structures depending on ionic conditions, as shown for repeats at human telomeres and oncogene promoters [4,5,6,7]. When G-quadruplex structures form in duplex DNA, the C-rich DNA strand complementary to G-quadruplex-forming sequences can form a four stranded i-motif at low pH [8], in which two tracts of cytosines form interdigitated C•C^+^ base pairs [7,9,10] (Figure 1C).

DNA sequences that can form G-quadruplex structures appear common in many genomes. G-quadruplex/i-motif-forming sequences occur at telomeres repeats in higher organisms, sequences from which G-quadruplex structures were first identified [11]. In humans, they can occur in immunoglobulin switch regions, oncogene promoters, the first introns of genes, and the 5’ untranslated regions near translation start sites [4,5,7,12]. These sequences also occur in bacterial genomes [13,14,15,16,17]. Reports vary as to the assessment of the potential for the participation of G-quadruplex structures in the regulation of gene expression in *E. coli* [13,14,18,19]. As demonstrated by Duquette et al., certain human sequences can form G-quadruplexes in *E. coli* [20], and G-quadruplex formation in mRNA can influence bacterial message utilization [21,22].

The existence of G-quadruplex DNA and RNA in bacteria is strongly suggested by the existence of proteins that can bind to, or interact with, these structures. In addition, Endoh et al. previously found at least five G-quadruplex-forming sequences in the ORF of *E. coli* genes [23]. Furthermore, at least 85 (G_3_T)_n_ repeats and 300 repeats similar to RET are found in K-12 (MG1655) *E. coli* genome. The plethora of proteins that can interact with these alternative structures strongly suggests that bacterial cells need to deal with the consequences of aberrant structure formation, or that various systems have employed the dynamic structure of DNA and RNA in a variety of biochemical transactions. G-quadruplexes can be remarkably stable and, like triplex DNA structures, can result in termination of DNA replication in vitro [24,25]. If these structures form in cells, they may block replication forks and provide substrates susceptible to genetic instability.

DNA helicases have been identified that can unwind replication-blocking DNA structures, including G-quadruplexes. These include the PifI helicase in bacteria and yeast where mutations in PifI, can lead to genetic instability of CEB1 G-quadruplex-forming repeats [26,27]. Many other bacterial proteins involved in maintaining the integrity of the genome have been identified that can bind to G-quadruplex DNA (or RNA) structures, including the filamentous bacteriophage fd gene 5 single strand binding protein [28], *E. coli* PolI [29], MutS [30]; helicases UvrD [31,32], RecQ family helicases [33], and others [34].

In *E. coli*, in addition to proteins and enzymes involved in genome maintenance, proteins involved in chromosome organization and compaction (nucleoid-associated proteins, NAP), which can often bind in a sequence nonspecific fashion, may also have the capacity to interact with alternative DNA conformations. The role of these proteins in DNA organization and packaging in chromosomes and large DNAs has been studied [35,36]. However, investigations into how proteins involved in chromosome organization may influence the equilibrium between canonical B-form DNA and alternative helical structures are lacking. Among NAPs, one important protein could play a role in DNA conformation, namely the Hfq RNA chaperone [37]. This protein, best known to interact with RNA, is a pleiotropic bacterial regulator that mediates several aspects of nucleic acid metabolism [37]. Hfq notably mediates translation efficiency by using stress-related small regulatory RNA (sRNA) and modulates the cellular levels of RNAs, either by changing their stability or through an unsolved transcriptional mechanism [37]. More recent analyses focused attention on the action of Hfq on nucleoid structure [35,38,39,40,41,42]. These analyses evidenced that Hfq cooperatively binds to DNA through a DNA:protein:DNA bridging mechanism, that it can form filaments on DNA, changing the mechanical properties of the double helix, with important implications for bacterial transcription and replication [35,36]. Here, we investigate the interaction of Hfq with quadruplex DNA. Our results suggest that G-quadruplex structures may form via several different pathways in cells, and that Hfq plays a role in their stability. Significantly, the formation of G-quadruplex structures during transcription and replication can lead to a dramatic increase in the rate of mutation associated with the repeat. The interaction with Hfq nucleoid-associated protein and its influence on DNA-quadruplexes structures may therefore be one additional mechanism responsible for modifying the rate of genomic evolution.

## 2. Materials and Methods

### 2.1. Bacterial Strains and Media 

The bacterial strains used include MC4100 and MC4100 *hfq::cm* [43] and BW25113 (*Δ(araD − araB)567, ΔlacZ4787(::rrnB-3), LAM^−^, rph-1, Δ(rhaD − rhaB)568, hsdR514)*. Luria-Bertani broth (LB) and K media [44] were supplemented with 30 μg/mL of ampicillin (Amp). For some Luria–Delbrück fluctuation assays, K medium was diluted with M9 + salts and thiamine to reduce the concentration of glucose and casamino acids. LB plates for Luria–Delbrück fluctuation assays for chloramphenicol resistance (Cm^r^) contained 25 μg/mL of Cm. Selection of tetracycline resistant (Tet^r^) revertants utilized LB plates containing 7.5 µg/mL of Tet for MC4100 derivatives. 

### 2.2. Cloning Quadruplex-Forming Repeats 

Complementary DNA oligonucleotides comprising DNA inserts (G_3_T)_4_, (G_3_T)_8_, and a RET oncogene G-quadruplex-forming sequence 5’-GGGGCGGGGCGGGGCGGGGGCG-3’ were chemically synthesized as EcoRI fragments (Integrated DNA Technologies, Coralville, IA, USA). These repeats form stable parallel G-quadruplex structures [10,45,46,47] (Figure S1). DNA sequences were hybridized and ligated into the *Eco*RI sites of plasmids pGEM^®^-Z3 (Promega, Madison, WI, USA) for structural studies and in the chloramphenicol acetyltransferase (*CAT*) gene in pBR325 for genetic analysis, as described previously [44]. For experiments with the *hfq* strains, the (G_3_T)_8_ and a RET oncogene sequences were synthesized and cloned into the *Bam*HI site in the *Tet* gene of pBR325, because the *hfq* strains are Cm^r^ [43]. The RET oncogene repeat, with duplicated *Eco*RI or *Bam*HI sites comprises 30-bp.

Cloned repeat DNA sequences were confirmed by DNA sequencing. To construct pBR235 plasmids, containing an inverted *Amp* gene and ColEI replication origin, purified pBR325 derivatives were simultaneously incubated with *Bsr*BI and T4 DNA ligase. Inversion of the *Bsr*BI fragment creates *Sac*I and *Sac*II sites. The inversion was confirmed by PCR analysis. Plasmids were purified by an alkaline lysis, CsCl-ethidium bromide density gradient protocol. 

### 2.3. Measurement of Cm^r^ or Tet^r^ Mutation Rates and Analysis of Revertants 

Plasmid pBR325 provides an excellent model for measuring rates of deletions of DNA sequences. Chloramphenicol resistant (Cm^r^) reversion in this genetic selection system reports complete or partial deletion, as well as simple frameshift mutations [44,48,49,50,51]. To ascertain potential differences in rates of instability when the G-rich strand comprises the leading or lagging strands of replication, the orientation of the unidirectional ColE1 replication origin and ampicillin gene was reversed creating pBR235-based plasmids. See Supplemental Figure S3.

Mutation rates were determined by Luria–Delbrück fluctuation assays [52]. An overnight culture, started from a single colony, was diluted to ~10^4^ cells/mL and eighteen parallel 1-mL cultures were then grown overnight to stationary phase. Viable cell counts were determined for three to six cultures by plating cell dilutions on plates containing ampicillin (or ampicillin and chloramphenicol for the *hfq* derivatives). The remaining cultures were used to determine the number of Cm^r^ or Tet^r^ revertants, by plating all cells on LB + Tet or LB + CAP plates. Mutation rates were calculated using the Ma–Sandri–Sarkar (MSS) estimator [53,54,55,56].

To determine the mutation spectrum, plasmid DNA was purified and transformed into HB101 to select a pure, individual plasmid, which was then purified and used for PCR and DNA sequence analysis. Platinum Taq Polymerase High Fidelity (Thermo Fisher Scientific, Waltham, MA, USA) was used for PCR analysis as the buffer (600 mM Tris-SO_4_, 180 mM (NH_4_)SO_4_, pH 8.9) is devoid of K^+^ that stabilizes G-quadruplex structures. Five μL reactions contained DNA in PCR reaction buffer (1× high fidelity PCR Buffer, 200 μM dNTPs, 0.5 μM BamL or Rep5, 0.5 μM BamR or Rep3, 1 U Platinum Taq DNA Polymerase High Fidelity, and 2 mM MgSO_4_). PCR conditions involved 30 cycles of 30 sec at 94 °C, 30 sec at 60 °C and 2 min at 72 °C. The primers were: BamL (5’GAAGCGATGAACCTCGGTGA3’) and BamR (5’GATCTTCCCCATCGGTGAT3’) for inserts cloned into the *Bam*HI site, and Rep 5 (5’GCACAAGTTTTATCCGGCCTTTATTC3’) and Rep 3 (5’GGGATAGTGTTCACCCTTGTTACAC3’) for inserts in the *Eco*RI site. The PCR products were separated on 5% polyacrylamide gels in TB buffer (40 mM Tris-Borate, pH 8.3). The length of the repeat remaining in the PCR products from the revertants was analyzed using Kodak ID Image Analysis software. 

### 2.4. Analysis of G-Quadruplex Structure Formation in Supercoiled DNA

Plasmid topoisomers were analyzed for the relaxation of superhelical turns using agarose-chloroquine gel electrophoresis in 1.75% agarose gels run in 40 mM Tris, 50 mM potassium acetate, 1mM EDTA, pH 8.3, containing sufficient chloroquine to resolve individual topoisomers. Transcription reactions were performed basically as described [20] using T7 or SP6 RNA polymerases (New England Biolabs, Ipswich, MA, USA) in RNAPol buffer supplemented with 0.5 mM ATP, UTP, GTP, and CTP, 40 mM KCl and 20 µg/mL RNaseA, for 1–2 hr at 37 or 40 °C. Samples were precipitated with two volumes of ethanol and redissolved and digested with RNaseA H (New England Biolabs, Ipswich, MA, USA) as recommended.

### 2.5. Binding Assays of Hfq to dG_7_

Hfq protein was purified as described previously [57]. The oligonucleotide dG_7_ was purchased from Eurogentec. The G-quadruplexes were prepared in water by heating at 95 °C for 5 min and then slowly cooling down to room temperature. We confirmed using native PAGE and SRCD that the quadruplexes form, even without salts. Fluorescence anisotropy measurements were collected as described previously [42]. For ATR-FTIR infrared spectroscopy, the complex was formed by adding the protein to the pre-formed parallel G-quadruplexes, lyophilized, and subsequently dissolved at a final oligonucleotide concentration of 2 mM in 20 mM Tris-HCl pH 7.6 containing 100 mM NaCl in D_2_O. ATR FT-IR spectra were recorded as described previously [42]. Note that the use of homo-oligomeric dG_7_ was mandatory for the FTIR analysis (introducing different bases as those found in RET or (G_3_T)_n_ would not allow precise analysis of H-bonding). Results were confirmed using synchrotron radiation circular dichroism (SRCD) with the same parallel quadruplex. For SRCD analysis, measurements and data collection were carried out on DISCO beam-line at the SOLEIL Synchrotron (proposals 20181037 and 20190015) [58]. 2–4 µl of samples were loaded into circular demountable CaF_2_ cells of 20 microns path length [59]. Chloride ions concentration was kept low (50 mM) to extend the spectral absorption edge to 175 nm, based on photomultiplier high tension (HT) cutoff. The protein concentration was 30 µM and that of dG_7_ 2mM. Three separate data collections with fresh sample preparations were carried out to ensure consistency and repeatability. Spectral acquisitions at 1 nm steps with 1.2 s integration time, between 320 and 180 nm were performed in triplicate for the samples as well as for the baselines. (+)-camphor-10-sulfonic acid (CSA) was used to calibrate amplitudes and wavelength positions of the SRCD experiment. Data-analyses including averaging, baseline subtraction, smoothing, scaling were carried out with CDtool [60].

## 3. Results

### 3.1. Formation of G-Quadruplex DNA in Supercoiled Plasmid 

The formation of a G-quadruplex in supercoiled DNA should result in the relaxation of superhelical turns, as occurs during formation of other alternative conformations, including cruciforms, Z-DNA, intramolecular triplex, unwound structures, and slipped strand DNA [1,50,61]. Once formed, alternative DNA structures are stable in supercoiled DNA allowing detection by measuring topoisomer relaxation on agarose gels. pGEM^®^-Z3 plasmids containing the G-quadruplex-forming repeats were incubated overnight at various temperatures (20–55 °C) in several low and higher ionic strength buffers (Tris or cacodylate), including 20 mM Tris, pH 7.6, and 100 mM KCl (or 100 mM NaCl) [62] to promote a structural transition. For all plasmids, at natural helical densities, no relaxation of supercoils indicative of a structural transition in supercoiled plasmid was observed (see for example Figure S2). This is in agreement with a comprehensive analysis of G-quadruplex formation in supercoiled DNA [63].

**Figure 2:**
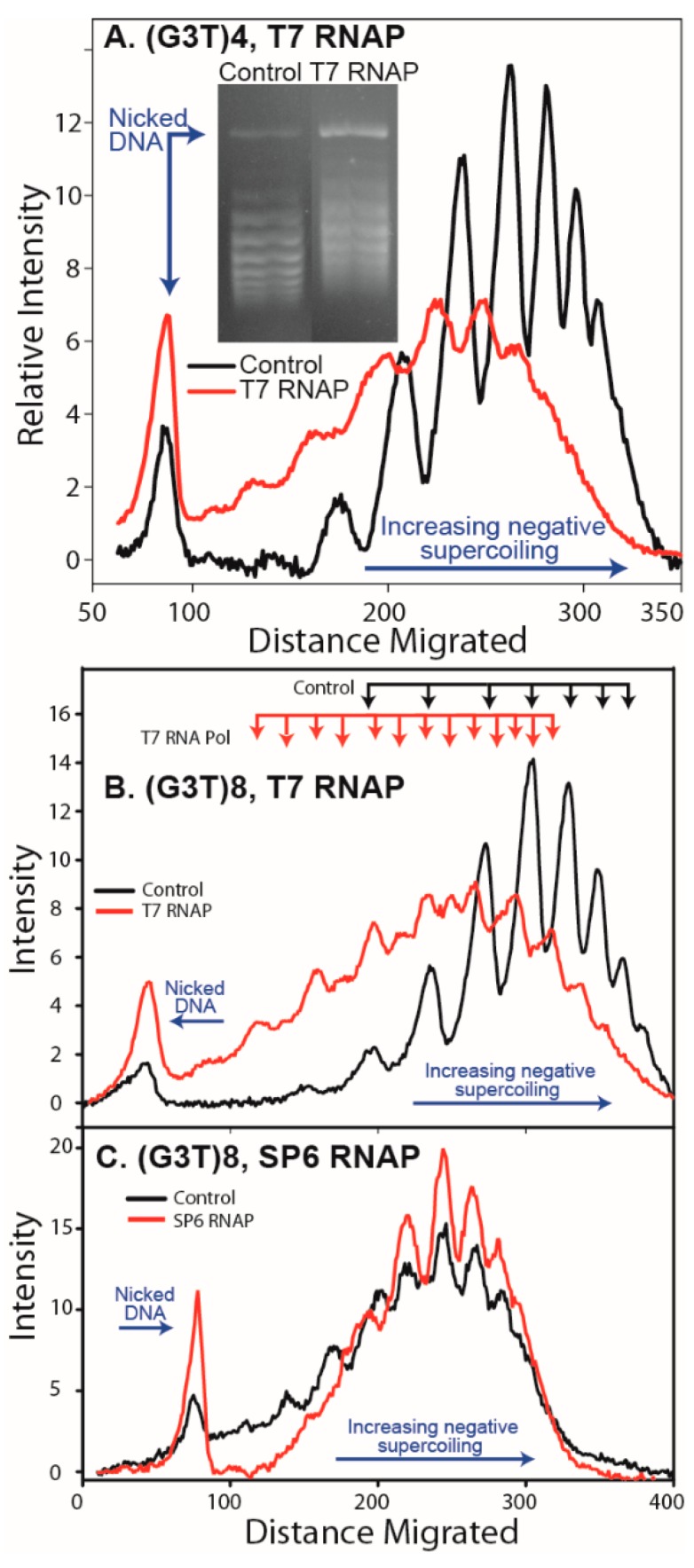
Formation of G-quadruplex during transcription. Plasmid pGem derivatives containing cloned G-quadruplex-forming repeats were incubated in RNA polymerase buffer with either T7 or SP6 RNA polymerase as described under Materials and Methods. Following treatment with RNaseH to remove any resulting hybridized RNA in a R-loop, individual topoisomers were resolved on an agarose gel containing chloroquine. (**A**) pGEM-(G_3_T)_4_ transcribed with T7 allows R-loop formation during transcription of a (G_3_U)_4_ containing mRNA. The inset shows an agarose gel. The scan shows positions of nicked DNA and direction of increasing superhelical density. A complete shift of the topoisomers to lower superhelical density (ΔL = −1.5) is evident. (**B**) Transcription of (G_3_T)_8_ with T7 RNAP in the direction where R-loop formation can occur shows a shift in superhelical density. (**C**) Transcription of (G_3_T)_8_ with SP6 in the opposite orientation, where R-loop formation should not occur, shows no shift.

G-quadruplex-forming repeats from immunoglobulin switch regions and certain oncogene promoters can form intramolecular quadruplex structures during transcription when the G-rich tract is transcribed into RNA that hybridizes to the C-rich DNA strand resulting in the formation of a G-loop (or R-loop) [20,64,65]. To determine if the (G_3_T)_4_, (G_3_T)_8_, and RET sequence could form G-quadruplex structures during transcription, T7 and SP6 RNA polymerase reactions using pGEM^®^-Z3 plasmids, containing the repeats cloned between convergent T7 and SP6 RNA polymerase promoters, were performed in the presence of KCl to stabilize G-quadruplex structures [20]. Plasmids were subsequently treated with RNaseH to remove any hybridized RNA strand. This should leave a stable G-quadruplex in supercoiled DNA and be detectable by the relaxation of superhelical turns observed on an agarose-chloroquine gel. Repeats containing the (G_3_T) motif formed G-quadruplex structures on transcription when the RNA contained the G-rich repeat, but not the C-rich repeat (Figure 2). For the 16-bp (G_3_T)_4_ repeat, a relaxation of 1.5 supercoils was observed, as expected for unwinding ~1.6 superhelical turns (Figure 2A). From the complete shift of the topoisomer distribution, the formation of G-quadruplex occurred in nearly 100% of topoisomers in the DNA sample. The (G_3_T)_8_ repeat exhibited a more complex gel pattern with relaxation overall of ~2.5 supercoils (Figure 2B). This is consistent with formation of two (G_3_T)_4_ G-quadruplex structures. Moreover, as the gel pattern shows double peaks at intervals of 0.5 turns, this may represent a mixture of plasmids with one or two G-quadruplex structures. The formation of stable G-quadruplex structures during transcription was not observed for the RET sequence or the (G_3_T)_8_ repeat when the RNA contained the C-Rich repeat (Figure 2C). 

### 3.2. G-Quadruplex Structures Promote Genetic Instability in E. coli 

**Figure 3:**
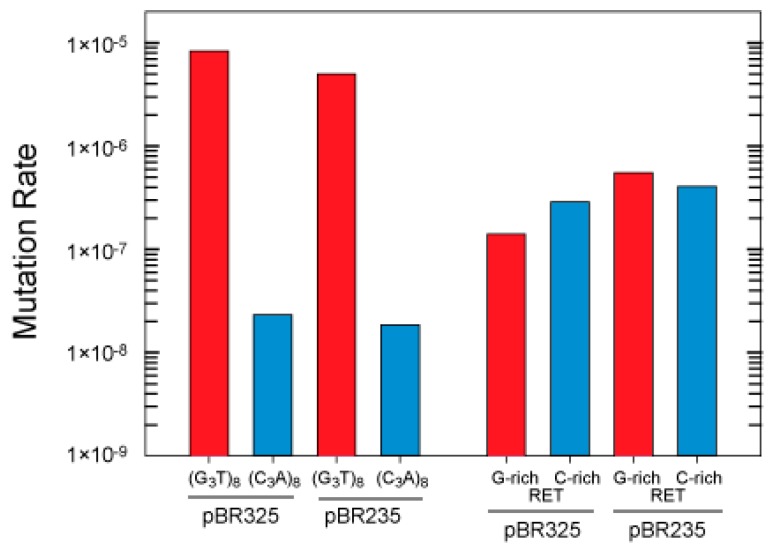
Effect of repeat orientation and leading or lagging strand placement on mutation rates in BW25113. Mutation rates for (G_3_T)_8_ and RET quadruplex sequences are shown. Mutation rates were determined using a Luria–Delbrück fluctuation analysis as described under Materials and Methods. The results are plotted for the G-rich or C-rich tract comprising the nontemplate (or coding) strand during transcription. The G-rich orientation allows the possibility of R-loop formation that can support G-quadruplex formation. Red bar, G-rich nontemplate strand; blue bar, C-rich nontemplate strand.

The (G_3_T)_8_ repeat was cloned in both orientations in the *CAT* gene in pBR325 to compare the effect on genetic instability of placing the G-rich strand in the nontemplate or template strand where transcription would or would not result in G-quadruplex formation, respectively (Figure S3). In the orientation in which G-quadruplex formation can occur during transcription (G-rich as nontemplate strand), the mutation rate in strain in BW25113 was 354 times higher than in the opposite orientation in pBR325 and 270 times higher in pBR235 (Figure 3). This dramatic difference is consistent with an interpretation that in one orientation, G-quadruplex structures form in cells leading to increased rates of instability. In contrast, the RET oncogene repeat, which did not show G-quadruplex formation on transcription, did not show a large difference in mutation rates when cloned in opposite orientations (less than 2-fold differences). A small effect of changing the direction of replication on mutation rates was observed with both inserts (1.6 and 1.3-fold for the (G_3_T_8_) repeat and 4 and 2-fold for the RET sequence, in the two orientations). 

### 3.3. Influence of Hfq on the Instability of Quadruplex-Forming Repeats 

**Figure 4:**
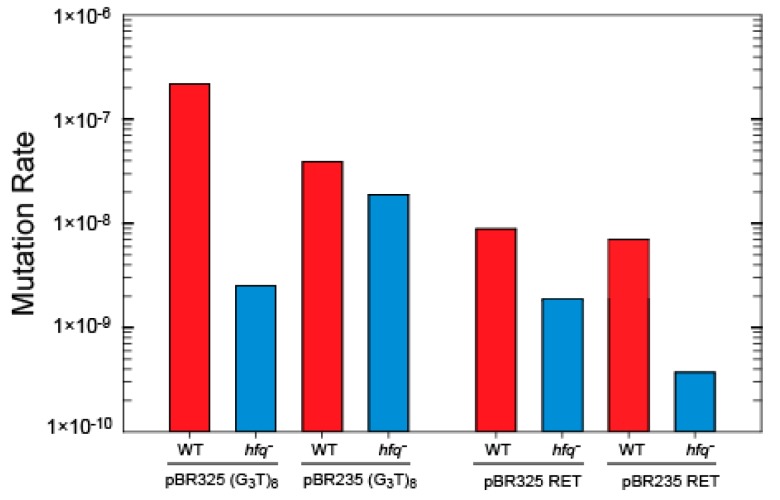
Effect of Hfq on mutation rates for (G_3_T)_8_ repeats in plasmids pBR325 and pBR235.

Mutation rates for the RET oncogene and (G_3_T)_8_ repeats, cloned into the *Bam*HI site in the *Tet* gene of pBR325 and pBR235 in the wild type strain (MC4100) and isogenic *hfq* mutant (MC4120) are shown (Figure 4). Use of the *Tet* gene as a mutational reporter, which reports only complete deletions, was necessary as MC4120 is Cm^r^, from transposon mutagenesis of the *hfq* gene [43]. Mutation rates for the RET oncogene and (G_3_T)_8_ repeats, in either orientation, were decreased in cells containing the deletion of *hfq.* Rates decreased 4.6 and 19.4-fold for the G-rich and C-rich leading strand orientations for the RET repeat, and 86 and 2.13-fold in the G-rich and C-rich leading strand orientations for the (G_3_T)_8_ repeat, respectively. 

### 3.4. Interaction of Hfq with G-Quadruplex DNA 

**Figure 5:**
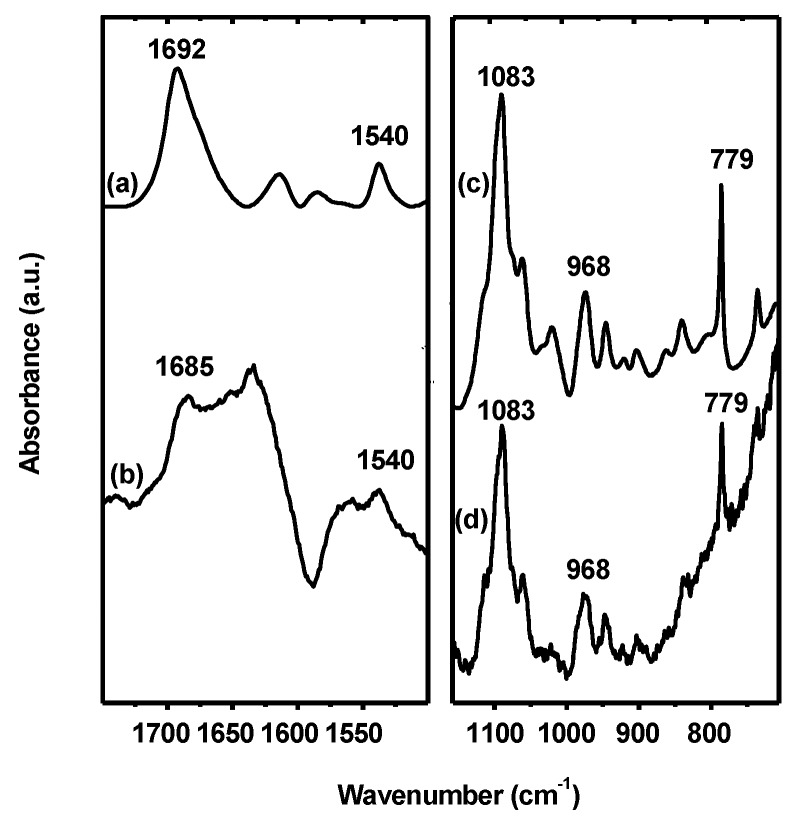
FT-IR analysis of G-quadruplexes in the presence or absence of Hfq. (**a**,**b**): FT-IR spectra are presented in the region of the in-plane double bond stretching vibration of the guanine. (**a**) Spectrum of parallel G-quadruplexes formed by four dG_7_ strands. (**b**) Difference spectrum of dG_7_ complexed with Hfq. The shift of the band from 1692 to 1685 cm^−1^ suggests that the parallel G-quadruplex is bound to Hfq and that the protein influence the stacking of the G-quadruplex quartets. The band at 1540 cm^−1^ is indicative of the presence of the Hoogsteen bond between N7 and N2-H. (c-d): FT-IR spectra in the region of the phosphate and sugar-phosphate backbone. (**c**) Vibrations of parallel G-quadruplex formed by four dG_7_ and (**d**) difference spectra of dG_7_ complexed with Hfq. Note that difference spectrum is always more noisy than the original spectrum. This subtraction is mandatory, however, as the protein and nucleic acid absorbance superimpose in important regions of the spectrum. The symmetric stretching vibration of the phosphate groups of the guanine strands at 1083 cm^−1^ indicates the presence of a parallel G-quadruplex structure.

While Hfq binds to DNA and RNA [41,66], its binding to G-quadruplex structures has not been investigated. Hfq:dG7 quadruplex complex formation was confirmed by EMSA and the equilibrium dissociation constant (Kd) of the complex was 1150 ± 110 nM as measured by fluorescence anisotropy (Figure S4). In parallel, interaction of Hfq and G-quadruplex structures and possible structural changes were analyzed by ATR infrared spectroscopy and circular dichroism. Duplex, triplex, and G-quadruplex structures can be identified by FT-IR spectroscopy by analysis of diagnostic absorption bands specific for various hydrogen bonding and base paring schemes [67]. We analyzed G-rich sequences dG_7_ by ATR FT-IR in the presence or absence of Hfq. Oligonucleotides dG_7_ form an intermolecular four-stranded parallel G-quadruplex [68]. Each guanine of the G-quadruplex is involved in two Hoogsteen hydrogen bonds with adjacent guanines of the G-quadruplex. H-bonds formed between C6=O6 and N1-H and N7 and N2-H groups (Figure 5) are observed on the FT-IR spectrum of parallel G-quadruplexes as bands corresponding to the carbonyl stretching at a wavelength just above 1690 cm^-1^ and an absorption band located around 1540 cm^−1^ indicative of the N7—N2-H hydrogen bond, respectively [68]. We clearly observe these two features in the spectra of dG_7_ (Figure 5) with carbonyl stretching band at 1692 cm^−1^, while it is observed at 1682 ± 3 cm^−1^ for antiparallel G-quadruplexes and at 1665 cm^−1^ for a free guanine carbonyl [68,69]. The spectrum of the protein was subtracted from that of the G-quadruplexes in presence of Hfq. In this difference spectra, the effect of Hfq on associated G-quadruplex can be observed on the C6=O6 carbonyl band, previously observed at 1692 cm^-1^ and now observed at 1685 cm^−1^ (Figure 5). This shift of the guanine absorption band suggests that, while the guanine carbonyl is still engaged in a hydrogen bond, the stacking of the parallel G-quadruplex has been perturbed [68]. Moreover, the band at 1540 cm^−1^, indicative of the presence of the N7-N2-H Hoogsteen bond, and the characteristic band at 1083 cm^−1^, corresponding to the symmetric stretching vibration of the phosphate groups of the guanine in G-quadruplex (Figure 5, [68]), are still present, indicating that G-quadruplex structures are not disrupted in the complex. Indeed, our data show that Hfq binding does not disrupt the characteristic H-bonds of the G-quadruplex quartet but leads to a modification of the stacking of the G-quadruplex quartets.

**Figure 6:**
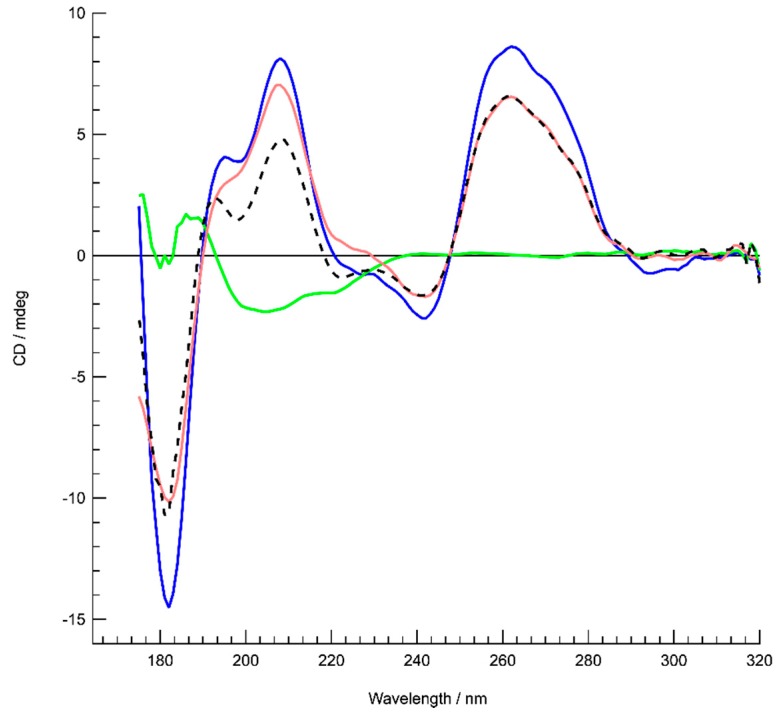
SRCD analysis of G-quadruplexes in the presence or absence of Hfq. Structure characterization of dG_7_ quadruplex complexed to Hfq by SRCD spectroscopy. Spectra of dG_7_ in the absence (red) and presence of Hfq (blue). Hfq alone (green). The spectrum of the complex (blue) is similar to the sum of the dG_7_ and Hfq spectra (dotted black), differing only in the strength of its amplitudes. This signifies most likely that upon complex formation an enhancement of already existing structural features in the dG_7_ quadruplex is occurring.

To confirm this result, circular dichroism spectroscopy was then used to see how the protein affects G-quadruplex structure. The G7 quadruplex alone produced a typical spectrum in accordance with previously reported parallel quadruplexes CD spectra [70]. The interaction with Hfq in the complex revealed stronger amplitudes without significant changes in the maxima and minima. As shown in Figure 6, in the region from 320–200 nm where the CD contributions originate from the nucleotide bases, sugars and phosphates in a general way, no particular changes, such as CD signal inversions or peak shifts were observed for the complex in comparison with the G7 quadruplex alone. This signifies that the overall quadruplex structure has been preserved and rather reinforced its structure upon interaction with Hfq. The increases of the amplitudes around 189 and 263 nm are most likely a result of increased G-G stacking [70] and Hoogsteen base pairing, respectively. These conclusions agree with the FTIR results. 

## 4. Discussion

The formation of alternative DNA conformations including quadruplexes may lead to mutations and genomic instability. We have investigated instability associated with G-quadruplex structure formation and the role of the RNA chaperone and DNA binding protein Hfq. Results presented indicate that G-quadruplex structures form in *E. coli*, especially during transcription when R-loop formation results in a single-stranded G-rich repeat. Specifically, the mutation rate for the (G_3_T)_8_ repeat was as much as 350 times higher when cloned in the orientation in which G-quadruplex structures can form during transcription, than when cloned in the orientation in which structures do not form. Structures may also form, albeit more rarely, in single-strand DNA during replication or during replication restart if forks pause near the G-quadruplex repeat tract. G-quadruplex structures may then promote subsequent genomic instability as they can block DNA replication or serve as sites for DNA repair activity. 

### 4.1. G-Quadruplex Structures and Pathways to Formation in Cells 

Several pathways are available for the formation of quadruplex structures in cells. A G-quadruplex and corresponding i-motif in the complementary strand could form in supercoiled DNA. While structures reportedly can form on denaturation and slow cooling supercoiled DNA [71], one report for a c-myc oncogene repeat has described formation during incubation at 37 °C in buffer containing K^+^ [62]. To date, however, we have not observed a transition in naturally supercoiled DNA under physiological conditions for the G-quadruplex-forming repeats analyzed here. Our results agree with a comprehensive analysis of role of DNA supercoiling in G-quadruplex formation [63] that shows a lack of formation of G-quadruplex by supercoiling alone. 

During transcription, an R-loop can form when a G-rich RNA strand hybridizes with the C-rich DNA template strand, displacing the G-rich DNA strand. This occurs in immunoglobulin switch regions and certain oncogene promoters containing G-rich repeats [20,64,65]. Duquette et al. reported the transcription-induced formation of G-quadruplex from immunoglobulin sequences in *E. coli* [20]. In fact, RNA-DNA hybrid formation during transcription of G-rich sequences may be a common phenomenon [72]. Our results demonstrate that G-quadruplex formation occurs with (G_3_T)_4_ and (G_3_T)_8_ repeats during transcription in vitro (Figure 2). When the (G_3_T)_8_ repeat was cloned in the orientation in which R-loop formation in vivo could promote G-quadruplex formation, a high mutation rate was observed. However, when the repeat was cloned in the opposite orientation, where G-quadruplex formation is not expected to occur, the mutation rate decreased ~350 fold. This result is consistent with the formation of G-quadruplex in cells during transcription. Once formed, a G-quadruplex within an R-loop might then be encountered by a replication fork. In plasmids pBR325 and pBR235, this would place the quadruplex in the leading and lagging template strands, respectively (Figure S3).

G-quadruplex formation on transcription was not observed in the RET sequences. In addition, we observed that several other simple G-quadruplex-forming sequences did not form G-quadruplex in the transcription assay, although conditions in all experiments were the same. The analysis by Duquette et al. [20] showed transcription-induced G-quadruplex formation for many G-quadruplex-forming repeats in supercoiled, relaxed, and even linear DNA. Formation in vitro may depend on several factors including repeat sequence, flanking sequence, and polymerization conditions. The absence of a strong orientation dependence on mutation rate increases for the RET sequence (as was seen for the G3T repeats) also suggests that G-quadruplex formation in this sequence may not form with high probability on transcription in our in vivo mutation assay system. 

G-quadruplex structures may also form during DNA replication. Formation may occur in the leading strand, although it is generally considered to remain predominantly duplex, or in the lagging strand when single stranded. Uncoupling of polymerase and helicase may generate a single strand region ahead of the replication fork, in which a G-quadruplex may form. Results in yeast demonstrate greater G-quadruplex repeat instability when the G-rich strand comprises the leading template, but only in Pif1 helicase deficient cells or when cells are grown in the presence of the G-quadruplex ligand Phen-DC_3_ [26]. A large leading/lagging strand mutation bias was not observed for G-quadruplex repeats, unlike results for other DNA repeats [44,48,49,51,73,74], suggesting that these short repeats may not preferentially form in one template strand during DNA replication. While G-quadruplexes may form in the leading or lagging strand at the replication fork, results clearly demonstrate formation dependent on transcription. 

### 4.2. Deletion of G-Quadruplex-Forming Repeats 

In our assay, various mutations can occur at G-quadruplexes, resulting in reversion to a Cm^r^ or Tet^r^ phenotype. These include complete deletion by primer template slippage between *Eco*RI (or *Bam*HI) sites flanking the quadruplex-forming repeats or primer template slippage within a repeat tract that restores the reading frame. Complete deletion between flanking restriction sites can occur with high frequencies when associated with alternative DNA structure formation, such as hairpins and cruciforms within inverted repeats [48,49,74], or hairpins within certain direct repeats with quasipalindromic symmetry (e.g., (CTG)•(CAG) repeats) [44,73]. Moreover, in the case of alternative DNA secondary structure formation (other than perfect palindromes), leading/lagging strand asymmetries associated with deletions and duplications are generally observed [44,48,49,51,73,74]. One working hypothesis is that the formation of a G-quadruplex structure will bring the flanking restriction sites into close proximity, block DNA polymerase, and favor primer template misalignment between restriction sites leading to complete deletion of the repeat. 

The (G_3_T)_8_ repeat underwent high frequencies of deletion of two (G_3_T) units in the *CAT* gene and complete deletions in the *tet* gene (which only reports complete deletions) when cloned in the orientation in which transcription may form the G-quadruplex structures. Deletion of two (G_3_T) repeats may occur readily by primer template misalignment after polymerase is blocked by a parallel quadruplex. Note that deletion of one G-quadruplex unit, to (G_3_T)_7_, which may occur frequently, would not result in a Cm^r^ phenotype and is not detectable in our assay.

Plasmids pBR325 and pBR235 have a different direction of unidirectional replication from the ColE1 origin, which is reversed in pBR235 derivatives. This changes the assignment of the G-rich or C-rich strands to the leading or lagging template (Figure S3). As shown previously, the potential for DNA secondary structure formation in the lagging template can promote deletion of repeats that can form hairpin structures by factors of 20–1000 [44,48,74]. Similarly, DNA secondary structure formation involving hairpins in the leading template strand can favor duplication mutations by a factor of 200 [51]. Leading-lagging strand asymmetries for the (G_3_T)_4_ and (G_3_T)_8_ repeats that form a very stable G-quadruplex were minimal in BW25113. The (G_3_T)_8_ repeat showed a <7-fold leading/lagging strand difference in MC4100. Different leading/lagging ratios for (G_3_T)_8_ in the different genetic backgrounds reflect strain differences as observed previously for mutation rates of (CAG)•(CTG) repeats [44,50,73,75]. G-quadruplex structures can form at high levels of 9%–18% in plasmids isolated from *E. coli*-containing mutations in RNaseH that can digest the RNA strand of an R-loop and RecQ, a helicase that can remove quadruplex structures [20]. In the strains used here, both RNAseH and RecQ are active. In yeast, a preference for deletions when the G-rich strand comprised the leading template strand was been observed in a *PifI* helicase mutant or in the presence of a G-quadruplex binding ligand [26]. As discussed previously, for the (G_3_T)_8_ repeat, transcription may be the major source of G-quadruplex formation in cells. G-quadruplex structures are likely forming when transcribed in different genes, both the *Tet* and *CAT* antibiotic resistance genes. 

We note that base mutation rates for the (G_3_T)_8_ repeat and the RET sequence were different in the two genetic backgrounds and mutation reporter genes. The RET base frequency was higher in strain BW2514 (Figure 3) than in the MC4100 background (Figure 4). This reflects differences in genetic background, and that the repeats are cloned into either the *Eco*RI or *Bam*HI sites in the *CAT* and *Tet* genes in the mutation selection vectors. Factors that influence base mutation rates may include gene sequences flanking the repeat, natural replication pausing through the gene, and interaction between replication and transcription machinery in vivo. The differences in mutation rates for the same repeats cloned into different sites in the plasmid illustrate the importance of the sequence environment of G-quadruplex-forming repeats in terms of the probability for mutation. Recent analyses of deletion of G-quadruplex across genome species supports the idea of evolutionary selection for loss of these repeats [76]. We have previously observed a similar evolutionary pressure on quasipalindrome correction to perfect inverted repeats in bacteria [77]. Clearly, symmetry elements in DNA sequence can be a major driver in genome evolution. 

### 4.3. A Mutation in hfq Increases Stability of G-Quadruplex Repeats 

Hfq is a post transcriptional regulator, which influences RNA structure [37]. However, it also binds to DNA [38,42] and, as shown here, Hfq binds to G-quadruplex. Therefore, the influence of the Hfq protein on G-quadruplex instability was analyzed. For the (G_3_T)_8_ and RET oncogene repeats, the mutation rates were reduced in the *hfq* strain in the MC4100 background. The rate was 85 times lower for (G_3_T)_8_ when the G-rich strand comprised the leading template and when RNA polymerase and the replication fork can collide. This decrease is consistent with an interpretation that, in wild type cells, Hfq could bind and stabilize a G-quadruplex structure, in accordance with its ability to help nucleic acid annealing [57], and this could favor structure accumulation and increase the rate of deletion mutagenesis. This is indeed confirmed here by our in vitro analysis using FTIR and SRCD spectroscopies. Hfq thus appears to have an additional activity in that it can bind and stabilize G-quadruplex structures that may promote genomic instability. These support an interpretation that the effect of Hfq on mutation rates for the (G_3_T)_8_ and RET oncogene repeats is structure specific, rather than a general pleiotropic and indirect effect. 

Mutations in Hfq can alter DNA topology and have pleiotropic effects in cells [35,39,41,43,78]. Hfq binds to sRNA and can alter mRNA translation or message stability [79] that can alter expression levels of many genes. Mutations in Hfq can notably alter levels of expression of mismatch repair (MMR) genes *mutS* and *mutH* [78]. Chen and Gottesman [80] recently demonstrated that Hfq is involved in the repression of *mutS* in stationary phase through direct binding within the mutS 5′ untranslated region and through interaction with sRNA. Thus, higher MutS levels in *hfq* mutants can repress stress-induced mutagenesis in stationary phase *E. coli* by reducing MMR-dependent mutations. In our mutagenesis experimental protocol, cells are grown to stationary phase by 18–24-h growth before plating. Thus, the mutations detected are not likely the result of adaptive or stress-induced mutation [81] resulting from lower levels of MutS (and occurring after several days in stationary phase). MutS has been reported to bind to DNA containing parallel G-quadruplex structures [30] (as has MutS", the human MutS analog [82]), although binding to the compact, stable (G_3_T)_4_ quadruplex used for mutational analysis herein has not been tested. Considering the results of Chen and Gottesman [80] and Ehrat et al., [30], alterations in MutS levels may also alter G-quadruplex mutation rates. MutS may bind to quadruplexes, either stabilizing them or initiating repair events that could increase instability. Mutation rates were decreased in the Hfq deficient strain, suggesting a direct role of Hfq in G-quadruplex stability.

Our working hypothesis is that the formation of a G-quadruplex predominantly during transcription, or formation in a leading or lagging strand during replication presents a block to DNA replication. Mutations may arise as cells negotiate resolution of this obstacle. Mechanism for removal include structure-specific helicases, and in the case of formation by R-loop stabilization, RNaseH. Our results suggest that Hfq increases the G-quadruplex-associated mutagenesis rate directly by binding and stabilizing the structure. The pleiotropic roles of the multifunctional Hfq protein and its effects on other genes involved in RNA repair and mutagenesis in both exponential and stationary phase reveal the complexity of evolutionary pressures on genome evolution. 

## 5. Conclusions

In conclusion, our results suggest that certain repeats can form G-quadruplex structures in *E. coli* cells, especially during transcription when the G-rich strand can be displaced by R-loop formation. Structure formation may then facilitate replication misalignment, presumably associated with replication fork blockage, thus promoting genomic instability. Significantly, results also evidence that the RNA chaperone and nucleoid-associated protein Hfq is directly involved in the genetic instability associated with these sequences, in addition to indirect effects via *mutS* repression. Hfq binds and stabilizes G-quadruplex structure in vitro, stabilizing G-quadruplex in cells and promoting G-quadruplex-associated mutations. These observations add to our knowledge of bacterial genome plasticity, in the context of further understanding the evolution of bacterial pathogens. Furthermore, our results reveal a complex interplay between regulators of bacterial DNA and RNA metabolism with possible important implications to understand how bacteria can alter their genotypes and adapt to their environment.

## Supplementary Materials

The following are available online at https://www.mdpi.com/2076-2607/8/1/28/s1, Figure S1: Circular dichroism spectra, Figure S2: Lack of plasmid supercoil relaxation following incubation at 37 °C or 60 °C, Figure S3: Organization of G-quadruplex-forming repeats cloned into pBR325 and pBR235, Figure S4: Hfq Binding Anisotropy and EMSA for d(G_7_)_4_.
