# Supplementary material for: Role of Hfq in Genome Evolution: Instability of G-Quadruplex Sequences in E. coli
Source: Microorganisms. 2019 Dec 22;8(1):28. doi: 10.3390/microorganisms8010028 (PMC7023247; doi:10.3390/microorganisms8010028)
Supplement: Supplementary file 1 [file microorganisms-08-00028-s001.pdf]

microorganisms
MDPI

SUPPLEMENTAL INFORMATION: Role of Hfq in Genome Evolution: Instability of G-quadruplex sequences in *E. coli*

Virali J. Parekh[1], Brittany A. Niccum[2], Rachna Shah[3], Marisa A. Rivera[3], Mark J. Novak[4], Frederic Geinguenaud[5], Frank Wien[6], Véronique Arluison[7,8] and Richard R. Sinden*[1]

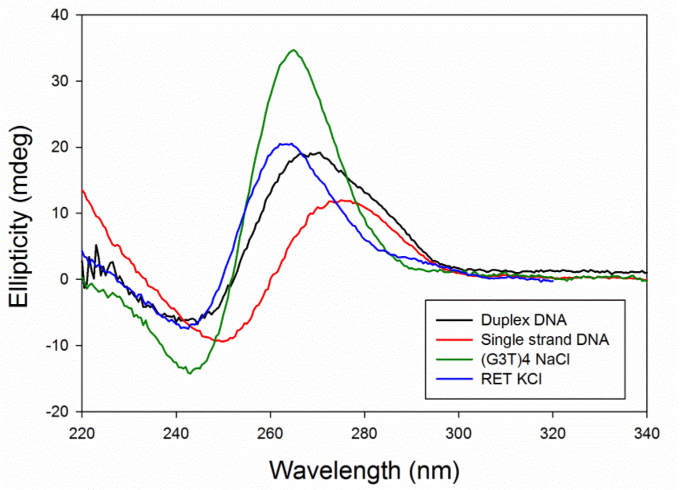

Figure S1. Circular dichroism spectra. CD spectra are shown for DNAs in 10 mM Na cacodylate, pH 7.0, and either 100 mM NaCl or 100 mM KCl. DNAs include duplex plasmid DNA (in NaCl), a synthetic single strand DNA with no potential for structure formation (in NaCl), a single strand oligos containing $(G_3T)_4$ in NaCl, and a single strand oligo containing the RET DNA sequence in KCl. The Tm's for the $(G_3T)_4$ repeat were 61°C for NaCl, >100°C for KCl and for the RET sequence 70°C for NaCl, 94°C for KCl.

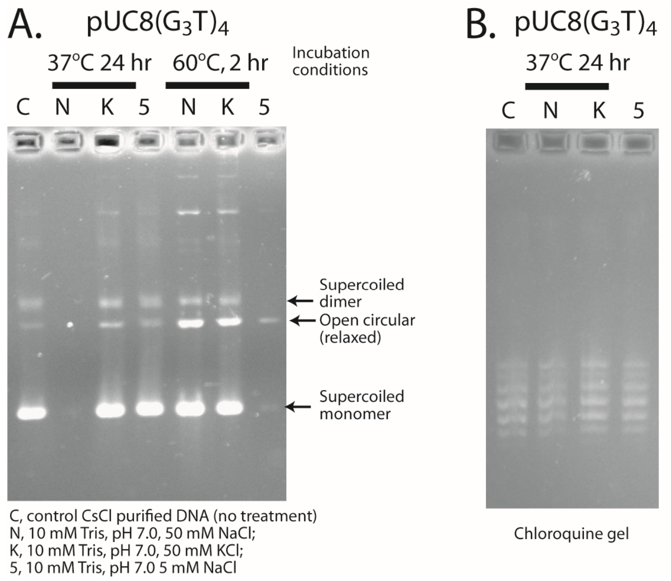

Figure S2. Lack of plasmid supercoil relaxation following incubation at 37°C or 60°C. A. CsCl-purified pUC8 plasmid containing (G3T)4 cloned into the EcoRI site was incubated at the temperatures, buffers, and times indicated and separated on a 1% agarose gel in TAE buffer (40 mM Tris, 50 mM potassium acetate, 1mM EDTA, pH 8.3). Formation of G-quadruplex structures would be expected to cause retardation in mobility of the supercoiled monomer. B. Chloroquine gel of samples incubated at 37°C showing no alternation in topoisomer distribution.

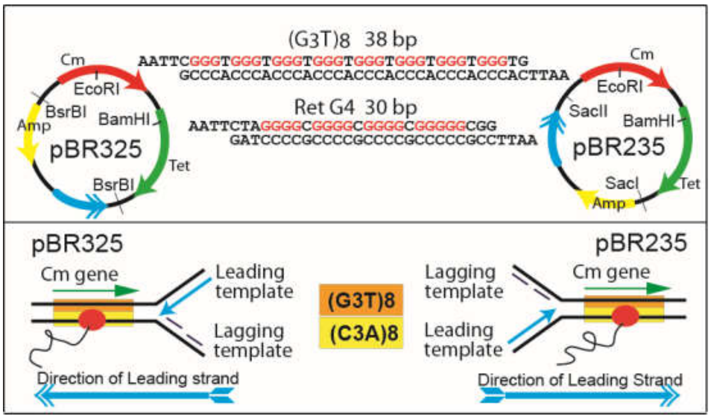

Figure S3. Organization of G-quadruplex-forming repeats cloned into pBR325 and pBR235. Top. Map of plasmids pBR325 and pBR235 showing the organization of the Chloramphenicol (Cm) gene and the BsrBI fragment containing the Amp gene and the ColE1 origin for unidirectional replication (ori ColE1). DNA repeat sequences are shown in the orientation cloned into the Cm gene. Bottom. The direction of replication is reversed in pBR325 and pBR235, such that the G-rich strand will comprise the leading or lagging template strands in either orientation, as illustrated for the $(G_3T)_8$ repeat.

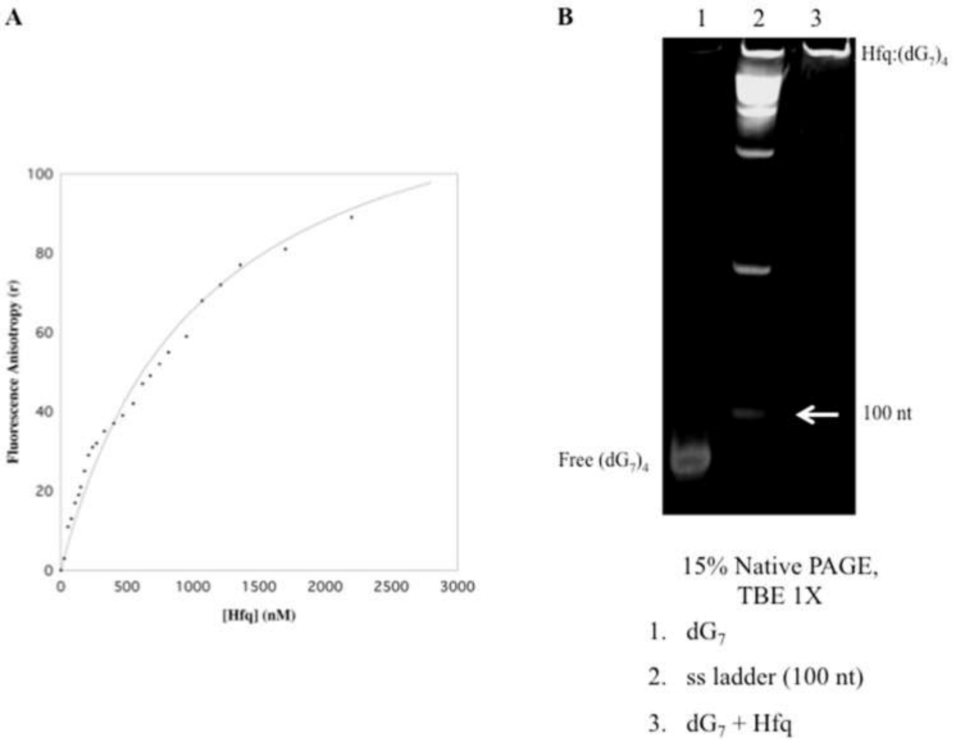

Figure S4. Anisotropy Binding curves and EMSA for d(G_7). *(A)* Fluorescence anisotropy measurements using 5′(fluorescein)-modified dG_7 were carried out as described in [41]. **(B)** Evidence of dG_7:Hfq complex formation was confirmed by EMSA. dG_7 (without dye) was stained with EtBr. dG_7 concentration was 0.5 μM, [Hfq] concentration was 10μM.
